# Symmetrical Triboelectric In Situ Self‐Powered Sensing and Fault Diagnosis for Double‐Row Tapered Roller Bearings in Wind Turbines: An Integrated and Real‐Time Approach

**DOI:** 10.1002/advs.202500981

**Published:** 2025-03-24

**Authors:** Song Wang, Xiantao Zhang, Tenghao Ma, Yun Kong, Shuai Gao, Qinkai Han

**Affiliations:** ^1^ State Key Laboratory of Tribology Department of Mechanical Engineering Tsinghua University Beijing 100084 China; ^2^ School of Mechanical Engineering Beijing Institute of Technology Beijing 100081 China; ^3^ State Key Laboratory of Mechanical Transmission College of Mechanical Engineering Chongqing University Chongqing 400044 China

**Keywords:** fault diagnosis, in situ self‐powered sensing, triboelectric nanogenerator, wind turbine

## Abstract

Double‐row tapered roller bearings (DTRBs) are widely used in wind turbines because of their high load‐bearing capacity and durability. However, wind turbines typically operate in harsh environments, subjecting bearings to complex working conditions, which significantly increases the difficulty of operational status monitoring. Traditional monitoring methods rely on external power sources and complex sensor networks, which make them susceptible to environmental interference, and complicated to maintain. This paper presents an innovative, integrated symmetrical single‐electrode triboelectric double‐row tapered roller bearing (SST‐DTRB) by incorporating a triboelectric nanogenerator (TENG) with DTRB. This scheme converts the frictional energy generated during bearing operation into electrical output, producing signals that enable simultaneous sensing of both ends of DTRB. Experimental results demonstrate that this monitoring scheme exhibits high sensitivity, stability, and reliability, with excellent robustness in material selection and design gap, and is capable of long‐term operation without external power sources. The effectiveness and self‐sensing capability of SST‐DTRB under variable speeds are validated using a wind turbine test bench. High‐accuracy bearing fault diagnosis under multiple conditions is achieved based on time‐frequency transformation and deep residual neural networks. The proposed SST‐DTRB provides in situ self‐powered sensing capability for wind turbines and offers new insights in the development of intelligent sensing systems.

## Graphical abstract

A novel symmetrical single‐electrode triboelectric double‐row tapered roller bearing is developed for in situ condition monitoring in wind turbines. The system converts operational friction into electrical signals for simultaneous sensing at both bearing ends. This self‐powered solution delivers high‐sensitivity fault diagnosis without external power sources, demonstrating exceptional reliability under variable speeds and providing a sustainable approach for intelligent monitoring.

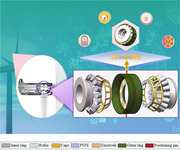

## 1 Introduction

As a promising renewable energy source, wind energy plays a crucial role in reducing greenhouse gas emissions and driving the transition to sustainable energy systems. As of 2023, the global installed capacity of wind power has exceeded 800 GW and is continuing to grow with technological advancements and increased investment in renewable energy infrastructure.^[^ ^1^, ^2^, ^3^, ^4^ ^]^ Wind turbines, the primary technology for converting wind energy into electrical energy, are critical for maximizing energy output and reducing operational costs.^[^ ^5^, ^6^, ^7^ ^]^ Double‐row tapered roller bearings (DTRBs) support wind turbine rotors and other rotating components, bearing both axial and radial loads while ensuring a smooth and reliable operation.^[^ ^8^, ^9^, ^10^ ^]^ DTRBs use two sets of tapered rollers and raceways positioned at the ends of the inner and outer rings. This design allows the bearing to withstand higher radial and axial loads while providing greater rigidity and stability. However, wind turbines operate in harsh environments with variable loads, extreme temperatures, and corrosive conditions, which pose significant challenges to the durability and performance of bearings. Effective monitoring of the bearing speed and condition is crucial for the early detection of wear, overheating, and other issues that can lead to catastrophic failures and costly downtime. Traditional monitoring methods, such as vibration analysis, temperature monitoring, and oil analysis, typically rely on external power sources and complex sensor networks.^[^ ^11^, ^12^, ^13^, ^14^ ^]^ These systems are expensive to install and maintain, and the need for regular maintenance and potential failures of the monitoring equipment limit their effectiveness.

In recent years, triboelectric nanogenerators (TENGs) have emerged as novel energy harvesting and mechanical sensing technologies.^[^ ^15^, ^16^, ^17^, ^18^, ^19^, ^20^ ^]^ TENGs convert the mechanical energy from friction into electrical signals based on the triboelectric effect and electrostatic induction principles. This technology offers advantages such as self‐powered sensing, high sensitivity, and no requirement for an external power source in mechanical sensing applications, thus enabling its operation in harsh environments.^[^ ^16^, ^21^, ^22^, ^23^, ^24^, ^25^ ^]^ TENGs can be integrated into bearings to monitor their operational status continuously. For example, Xie proposed a non‐contact triboelectric bearing sensor (NC‐TEBS) for monitoring the speed and skidding of deep‐groove ball bearings.^[^ ^26^ ^]^ This sensor had a charge replenishment device to increase the surface charge density and ensure output signal strength, achieving a maximum error rate of 0.25% in speed detection. Han designed a rolling free‐standing mode TENG (RF‐TENG) by adhering flexible interdigital electrodes to the outer ring of rolling bearings, avoiding direct contact between the flexible electrodes and rolling elements while maintaining the structural integrity of the bearing.^[^ ^27^ ^]^ While these solutions initially achieved TENG‐based bearing monitoring, they were mainly limited to single‐row bearings or single‐side monitoring, unsuitable for the separable symmetric structure of DTRBs, and unable to simultaneously monitor the operational status of both ends of the bearings. The unique structural design of DTRBs, featuring two rows of tapered rollers arranged in opposite directions, creates distinct operational dynamics at each end. This arrangement enables DTRBs to handle complex combined loads but also makes them susceptible to differential wear patterns and failure modes between the two rows. In wind turbine applications specifically, asymmetric loading from turbulent wind conditions and mechanical imbalances frequently results in different stress patterns at each end of the bearing. The two sides of a DTRB may face different working environments and load conditions, thus requiring the designed TENG to simultaneously monitor the operational status of both ends. Traditional TENG solutions are more suitable for single‐side monitoring and may overlook important information from the other side, leading to an incomplete assessment of the bearing condition.

Therefore, this study aims to develop an innovative and integrated symmetrical single‐electrode triboelectric DTRB (SST‐DTRB) for integrating a TENG with a DTRB. By converting the frictional energy generated during bearing operation into the TENG output, electrical signals can be produced to enable simultaneous self‐monitoring of both ends of the bearing. The experimental results demonstrate that this monitoring scheme exhibits high sensitivity, stability, and reliability, with excellent robustness in material selection and design clearance, and is capable of long‐term operation without an external power source. The self‐powered sensing and fault diagnosis capabilities of the proposed SST‐DTRB under variable speeds are validated using a wind turbine test bench with a transmission system structure consistent with actual wind turbines. By utilizing the short‐time Fourier transform (STFT), the multi‐channel outputs of the SST‐DTRB are synchronously converted into multi‐dimensional time‐frequency spectrograms containing spatial information. Based on deep residual neural networks, a bearing fault diagnosis is achieved with an accuracy of up to 95.6% under multiple operating conditions. The proposed SST‐DTRB presented in this study provides in situ self‐powered sensing and fault diagnosis capabilities for wind turbines. The key innovations include:

1. Structural innovation: An integrated self‐powered TENG‐based sensing scheme is designed that is suitable for the unique separable symmetric structure of DTRBs. This scheme features a compact structure and high sensitivity and is capable of long‐term operation without an external power source.
2. In situ self‐powered sensing and validation based on the wind turbine test bench: The effectiveness and reliability of the proposed SST‐DTRB are validated using a wind turbine test bench with a transmission system structure consistent with actual turbines. The SST‐DTRB achieves in situ speed monitoring and effective tracking of the stability of wind turbine operation.
3. High‐precision intelligent diagnosis: High‐accuracy bearing fault diagnosis is achieved under multiple operating conditions, based on time‐frequency transformation and deep residual neural networks, with an accuracy of up to 95.6%.
4. Application prospects: By addressing these challenges and demonstrating the effectiveness of the proposed scheme, this study provides new insights for the widespread application of TENGs in wind turbines.

## 2 Structure and Working Mechanism of SST‐DTRB

**Figure 1:**
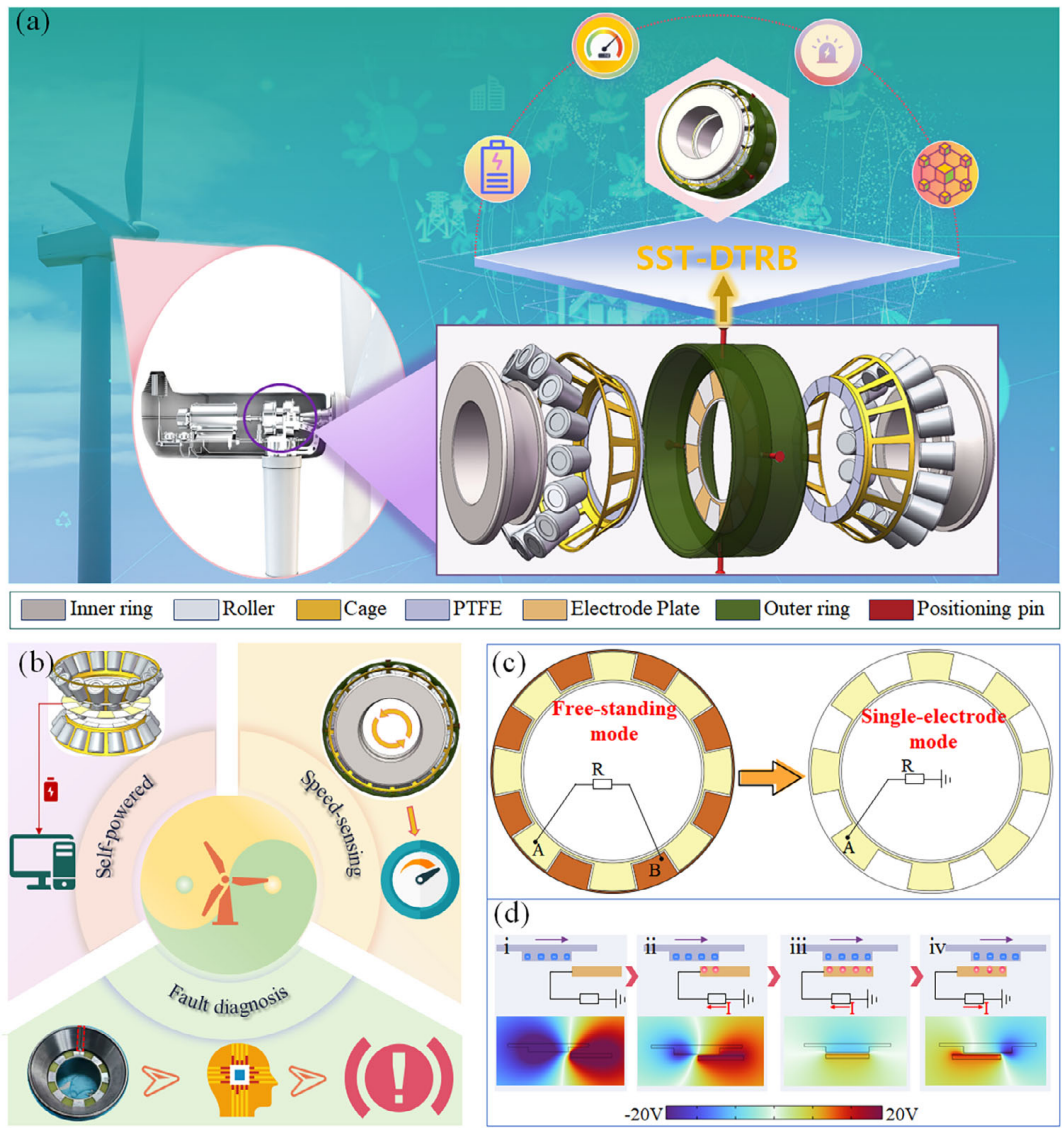
Application diagram and architecture of SST‐DTRB: a) application diagram and structure, b) key advantages, c) comparison between free‐standing layer mode and single‐electrode mode, d) working principle of SST‐DTRB.

Applying DTRBs to wind turbines requires bearings to withstand high radial and axial loads while operating stably in complex and harsh environments for extended periods. This study presents the design of an SST‐DTRB to achieve real‐time monitoring of DTRBs. As shown in **Figure** **1**a, the structure of the SST‐DTRB mainly consists of an inner ring, outer ring, rollers, cage, PTFE dielectric layer, electrode plates, and positioning pins. This design innovatively combines a TENG with a traditional DTRB structure to achieve integrated self‐powered, self‐sensing, and fault diagnosis functionality for DTRBs. Notably, utilizing the unique separable symmetric structure of DTRBs, the electrode plates are mounted on the outer ring and fixed with positioning pins. Two dielectric rings are attached to the end faces of the cage and rotate with them, periodically rubbing against the electrode plates to generate TENG electrical signals. The dominant frequency *f_v_* of an electrical signal can be expressed as

(1) $$f_{v}=N_{p}\cdot f_{cage}$$

where *N*_*p*_ represents the number of electrodes, and *f*_*cage*_ represents the cage rotation frequency.

The cage rotation frequency *f*_*cage*_ of the bearing^[^ ^28^ ^]^ can be expressed as

(2) $$f_{cage}=\frac{1}{2}f_{in}\left(1-\frac{d_{\min}+d_{\max}}{2D}\cos\alpha \right)$$

where the *f*_in_ is the output shaft speed, *d*_min_ represents the small end diameter of the roller, *d*_max_ represents the large end diameter of the roller, *D* represents the pitch circle diameter of the bearing, and α is the contact angle of the tapered roller. Evidently, the output electrical signal of the proposed SST‐DTRB contains rich characteristic components, with its dominant frequency closely related to the actual rotation frequency of the wind turbine and the operational status of both ends of the DTRB.

Figure 1b shows that the SST‐DTRB has three core functions: self‐powering, self‐sensing, and fault diagnosis. The self‐powering function allows the SST‐DTRB to utilize the frictional energy generated during bearing operation, enabling continuous sensing without requiring an external power source for the sensing component. The self‐sensing function allows the bearing to monitor its operational status in real‐time, outputting feedback that separately reflects the operational status at both ends of the DTRB. The two sides of a DTRB may experience different working environments and load conditions, and simultaneous monitoring of both ends provides comprehensive feedback on the operational status of the DTRB and wind turbine. More importantly, the SST‐DTRB possesses powerful fault diagnosis capabilities and can promptly detect and identify potential bearing faults, providing a reliable basis for preventive maintenance. The SST‐DTRB solves the problems of traditional bearing monitoring systems that rely on an external power source and achieves real‐time self‐sensing of the bearing conditions. This integrated design significantly improves the reliability and intelligence of wind turbine transmission systems. The integration of self‐powered, self‐sensing, and fault diagnosis functions provides a new technical approach for the intelligent operation and maintenance of wind turbines.

As shown in Figure 1c, the SST‐DTRB adopts a single‐electrode mode TENG. Compared with traditional free‐standing mode TENGs, the single‐electrode mode TENG reduces the number of wires by half, thereby lowering the complexity of installation and maintenance. The operating principle of the SST‐DTRB can be divided into four stages, as illustrated in Figure 1d. In the first stage, the dielectric ring protrusion separates from the electrode, and no charge is generated on the electrode. In the second stage, the protrusion partially aligns with the electrode, and positive charges accumulate on the electrode plate owing to electrostatic induction. At this time, current flows from the ground terminal to the electrode plate. In the third stage, the protrusion fully aligns with the electrode, and the potential difference between the electrode plate and the ground terminal reaches its maximum. In the fourth stage, the protrusion begins to leave the electrode area, and the positive charges on the electrode plate flow back to the ground terminal, producing a current from the electrode plate to the ground terminal. This cycle is repeated continuously to generate a sustained electrical signal output. The COMSOL simulation results intuitively demonstrate the charge distribution and electric field changes at each stage, which is consistent with the simulation results of other typical single‐electrode TENGs, thus validating the working principle of the SST‐DTRB.

## 3 Main Parameters Analysis and Output Performance of SST‐DTRB

**Figure 2:**
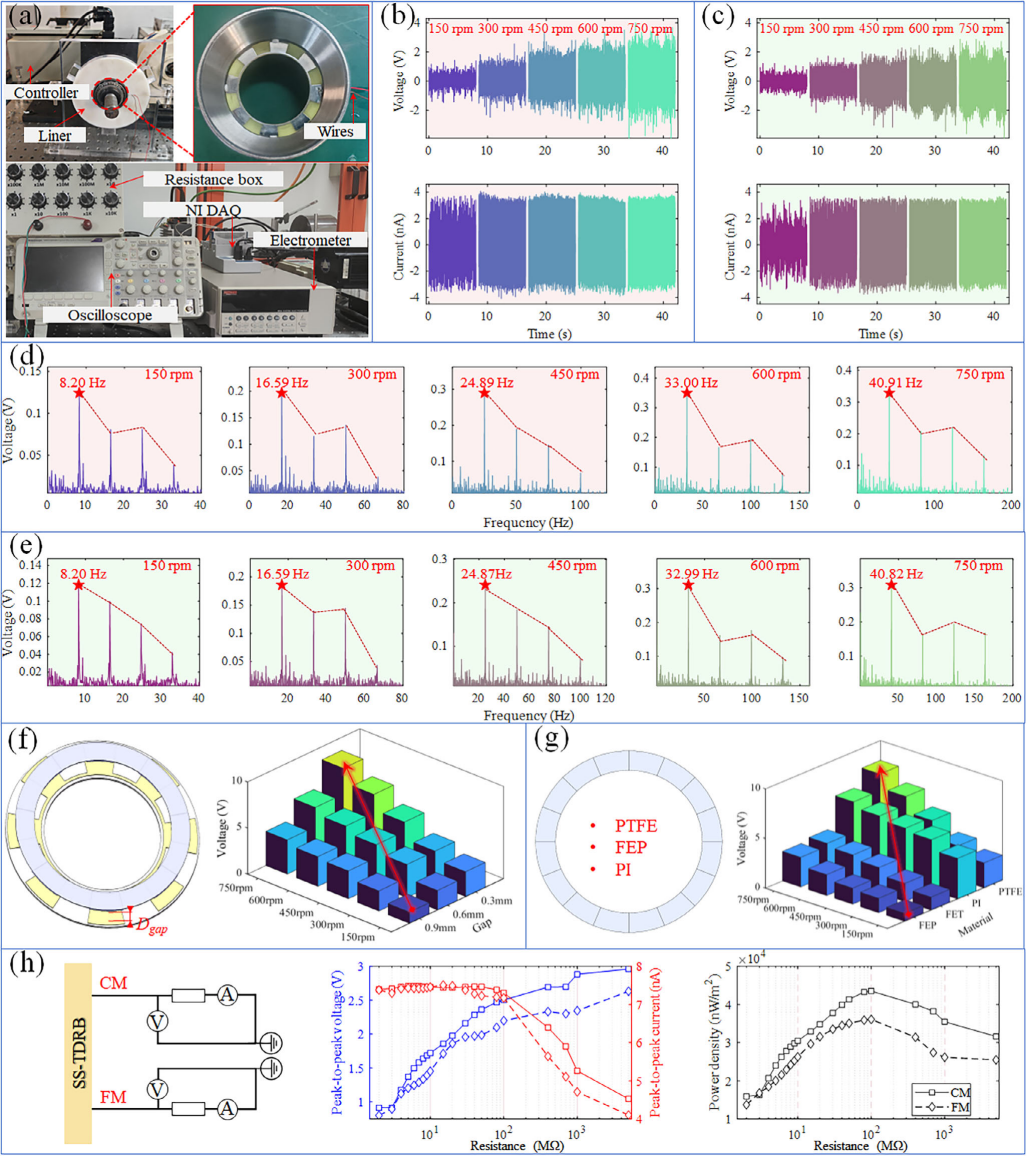
Output characteristics: a) SST‐DTRB test system, b) output on CM side and c) FM side, d) frequency spectrum on CM side and e) FM side, f) gap between dielectric ring and electrode plate, g) material of dielectric ring, h) voltage and power density under different resistances.

This section presents a series of detailed experiments and analyses to comprehensively evaluate the performance characteristics and applicability of the SST‐DTRB. **Figure** **2**a shows the SST‐DTRB test platform, which mainly consisted of a DC servomotor, coupling, frequency converter, bearing pad, and SST‐DTRB. The test instruments included an electrometer, National Instruments (NI) data acquisition card, resistance box, and oscilloscope to ensure the accuracy and comprehensiveness of data acquisition.

The effects of speed variation from 150 to 750 rpm on the voltage and current of the close‐to‐motor (CM) and far‐from‐motor (FM) sides of the SST‐DTRB are shown in Figure 2b,c, respectively. The results show that as the speed increased, the output voltages of both the CM and FM ends exhibited a continuous upward trend, whereas the peak current first increased slightly from 150 to 300 rpm and then remained relatively stable at higher speeds. This is because the voltage rise with speed is mainly affected by the increased frequency of contact‐separation between the electrode and friction layer, causing more frequent potential difference changes; while the current increases slightly at low speeds with increased contact frequency, but at high speeds, charge density reaches saturation and contact time shortens, limiting complete charge transfer and resulting in stable peak values. This phenomenon is consistent with reference,^[^ ^29^ ^]^ confirming the effectiveness of the SST‐DTRB at different operating speeds. Meanwhile, the slight differences in the output (amplitude and waveform) between the CM and FM ends were due to the differences in the operational details. To further analyze the output characteristics of the SST‐DTRB, a spectral analysis was performed on the output voltages of the CM and FM ends at different speeds using a fast Fourier transform (FFT), as shown in Figure 2d,e. The spectral analysis revealed the main frequency components of the output signal, which were directly related to the rotational speed of the bearing. The observed dominant frequencies corresponded well with the theoretically calculated values (Equation 1), confirming that the SST‐DTRB accurately reflects the motion state of the bearing. Due to the time‐varying nature of the contact state, the harmonic and modulation components of the dominant frequency appeared in the signal spectrum, providing detailed information on the rotation state. For example, the harmonics of *f_v_* can significantly increase because of rotor imbalance, misalignment, mechanical looseness, and structural resonance. The difference between the actual and theoretical values of *f_v_* represents the cage skidding, which may be caused by bearing faults or uneven loads. The output of the SST‐DTRB contains rich characteristics that provide vital support for self‐sensing.

Considering the possible axial motion in practical applications, the effect of the initial installation gap between the dielectric ring and electrode plate at the FM end on the output was investigated, as shown in Figure 2f. The results show that appropriately reducing the installation gap improved the output. However, maintaining a particular installation gap helps to avoid excessive wear of the TENG, which is essential for the practical application and long‐term reliability of SST‐DTRBs. The choice of the dielectric ring material significantly affected the performance of the TENG. As shown in Figure 2g, the effects of different materials (PTFE, FEP, PET, and PI) on the output voltage at the FM end were compared, maintaining a 0.3 mm installation gap between the dielectric ring and electrode plate. The results show that the SST‐DTRB achieved the maximum output voltage using PTFE, although its output was slightly weaker than that of the PI at low speeds. However, the excellent corrosion resistance, self‐lubrication, and high‐ and low‐temperature resistance make PFTE a high‐performance material widely used in multiple fields. Therefore, PTFE was selected as the dielectric ring material. Finally, the output characteristics of the SST‐DTRB were comprehensively evaluated by connecting the external resistances between the CM end, FM end, and ground terminal. As shown in Figure 2h, both CM and FM ends achieved maximum output power densities of 4.35 × 10^8^ and 3.61 × 10^8^ nW m^−2^, respectively, when the external circuit resistance was 100 MΩ. This method was used to analyze the power output characteristics under different load conditions, thereby providing an essential basis for circuit design in practical applications of SST‐DTRBs.

**Figure 3:**
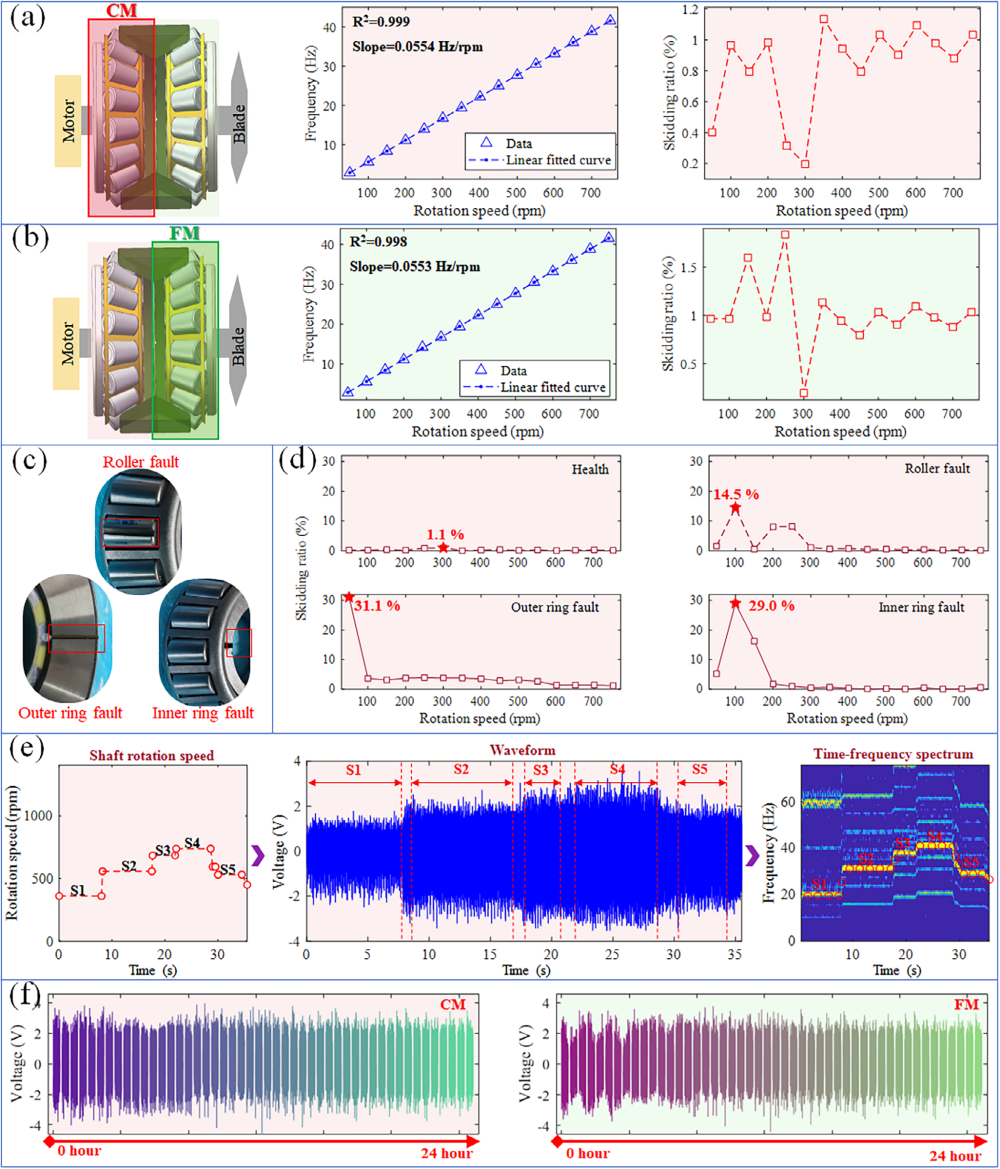
Condition monitoring capability and durability verification: a) linear relationship between signal frequency and rotation speed and monitoring of skidding ratio on CM and b) FM side, c) physical picture of faulty parts, d) affect of faults on skidding ratio, e) speed tracking capability during variable speed operation, f) durability verification.

Further experiments validated the ability of the SST‐DTRB to monitor operating conditions under a broader range of working conditions. **Figure** **3**a,b demonstrate the speed detection capability of the SST‐DTRB at 50–750 rpm. The experimental results show excellent linear relationships between the frequency and speed of the CM and FM ends. The coefficient of determination R^2^ for the fitting curves of the CM and FM ends reached 0.999 and 0.998, respectively, indicating the high‐precision speed detection capability of the SST‐DTRB. When the monitored cage rotation frequency *f_c_* = *f_v_*/*N_p_* deviates significantly from the theoretical value, cage skids may damage the cage, rollers, and raceways. The skidding ratio of the cage can be expressed as

(3) $$w_{s}=\frac{\left|{\hat{f}}_{c}-f_{c}\right|}{{\hat{f}}_{c}}\times 100\%$$

The skidding conditions at both ends under various speeds were studied by calculating the skidding ratio at different speeds. The results indicate that the CM and FM ends exhibited different skidding characteristics. This difference may stem from the uneven load distribution and slight differences in installation errors. Notably, at 250 rpm, the skidding ratio of the FM end reached a maximum of 1.8%, which is relatively small. To assess the sensitivity of the SST‐DTRB to bearing faults, the skidding situations of healthy and three typically faulty bearings at different speeds were compared at the CM end. As shown in Figure 3c, the roller, outer race, and inner race faults were selected for this study as they represent the most common and critical failure modes in DTRB, each generating distinct characteristic components that can validate the self‐sensing capability of SST‐DTRB. The experimental results (Figure 3d) clearly show that the introduction of faults significantly increased the skidding ratio of the bearing. Specifically, the maximum skidding ratios under the roller, outer ring, and inner ring fault conditions reached 14.5%, 31.1%, and 29%, respectively, which were significantly higher than those of healthy bearings. It is worth noting that bearing skidding and bearing wear exhibit an interconnected relationship, where bearing faults affect operational stability leading to skidding, while skidding disrupts pure rolling and accelerates wear. The distinct skidding patterns and spectral characteristics from different fault types demonstrate the system's potential for distinguishing between various fault conditions. This finding confirms the potential of the SST‐DTRB for the early detection and differentiation of bearing faults.

Figure 3e shows the performance of the SST‐DTRB under variable speed conditions. The variable speed tracking capability was verified by adjusting the input shaft speed. The actual speed change curve is in good agreement with the time‐frequency spectrogram obtained by STFT, demonstrating that SST‐DTRB can accurately capture dynamic changes in the bearing speed. Finally, to evaluate the long‐term reliability, a 24‐h continuous operation test was conducted (Figure 3f). During the test, data were collected from the CM and FM ends every 0.5 h. The results show that the output waveform of the SST‐DTRB remained stable during long‐term operation with no significant attenuation in the peak voltage. This result confirms the excellent durability of the SST‐DTRB, supporting its long‐term reliability in practical industrial applications. In summary, the detailed experimental results not only verify the excellent performance of the SST‐DTRB in speed detection, condition monitoring, and durability but also lay a solid foundation for its practical application in wind turbine bearing monitoring. The high precision, strong reliability, and powerful fault detection capabilities demonstrated by the SST‐DTRB make it a powerful tool for the preventive maintenance and intelligent monitoring of wind power generation systems.

## 4 Application of SST‐DTRB in Wind Turbine Test Bench

**Figure 4:**
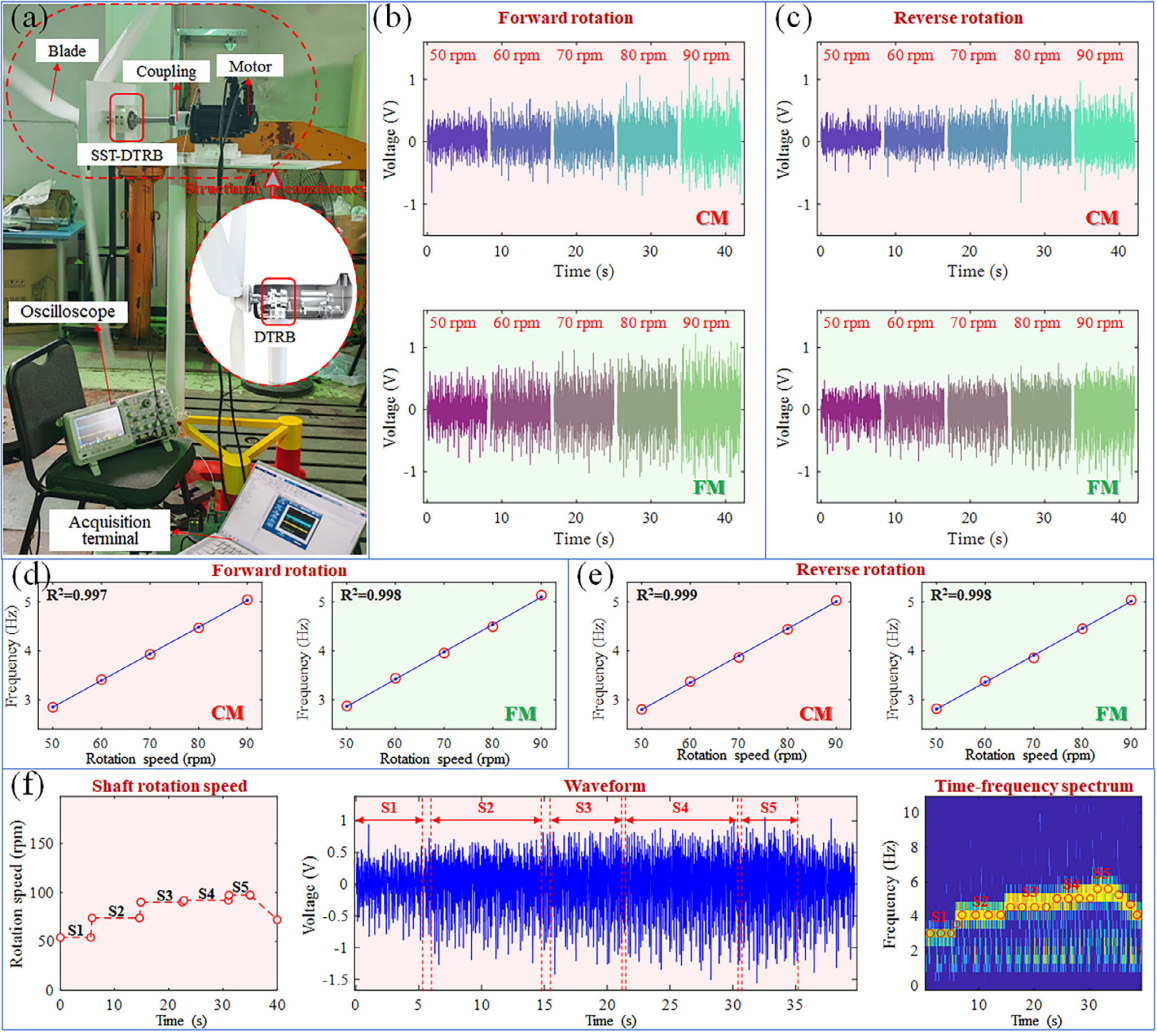
Application in wind turbine test bench: a) wind turbine test bench, b) outputs when forward and c) reverse rotation, d) speed fitting when forward and e) reverse rotation, f) speed tracking capability during variable speed operation.

A detailed wind turbine test bench was simulated to comprehensively evaluate the performance of the proposed SST‐DTRB in a realistic wind turbine environment. As shown in **Figure** **4**a, the drivetrain structure of the test bench closely emulated the characteristics of an actual wind turbine, thereby ensuring the validity of the test results. The core components of the test bench included blades, SST‐DTRB, couplings, and drive motors. Unlike actual wind turbines, this test bench used a motor to drive the blade rotation, allowing precise control and simulation of various operating conditions for more comprehensive and detailed performance evaluations. The SST‐DTRB was strategically installed near the blades, providing critical support and conforming to the drivetrain structure of actual wind turbines.

Figure 4b,c show the output characteristics of the CM and FM ends of the SST‐DTRB during forward rotation (clockwise rotation of the blades when viewed from the electrode direction) and reverse rotation, respectively. The experimental results clearly show that, as the rotational speed increased from 50 to 90 rpm, the output voltage amplitudes at both the CM and FM ends exhibited a significant increase in both rotational directions. The similar output characteristics observed during both forward and reverse rotation demonstrate that the SST‐DTRB can effectively function regardless of the rotation direction, which is particularly valuable for ensuring monitoring reliability during various operational modes of wind turbines. For a more detailed analysis of the SST‐DTRB performance, further verification of the rotational speed detection from the output signals was conducted. Figure 4d,e illustrates the relationship between the signal‐derived rotational frequency and actual rotational speed during forward and reverse blade rotations. The results indicate a strong linear correlation between the dominant frequencies of the CM and FM ends and the bearing rotational speed, with R^2^ >0.997. This confirms the ability of the SST‐DTRB to accurately detect wind turbine operational speeds and reveals that subtle differences in the signal characteristics between the CM and FM ends may reflect the varying conditions at the two ends of the bearing, providing an essential basis for detailed operational state monitoring. Moreover, the performance of the SST‐DTRB was further validated under dynamic conditions using variable speed tests. As shown in Figure 4f, the shaft speed was gradually increased from ≈50 to 100 rpm during the test and then reduced to ≈70 rpm. The waveform and time‐frequency spectrum of the SST‐DTRB output signal clearly reflected this speed variation process. The main frequency component in the time‐frequency spectrum accurately tracked the dynamic changes in the shaft speed (marked with red circles in Figure 4f), demonstrating the excellent capability of the SST‐DTRB in capturing dynamic variations in wind turbine bearing speeds. The comprehensive experimental results strongly confirm the efficiency and reliability of the SST‐DTRB in practical wind turbine environments. Its exceptional performance is evident not only under steady‐state conditions but its strong adaptability under dynamic variable speed conditions is also demonstrated, providing a solid technical foundation for the real‐time monitoring and fault diagnosis of wind turbine bearings.

**Figure 5:**
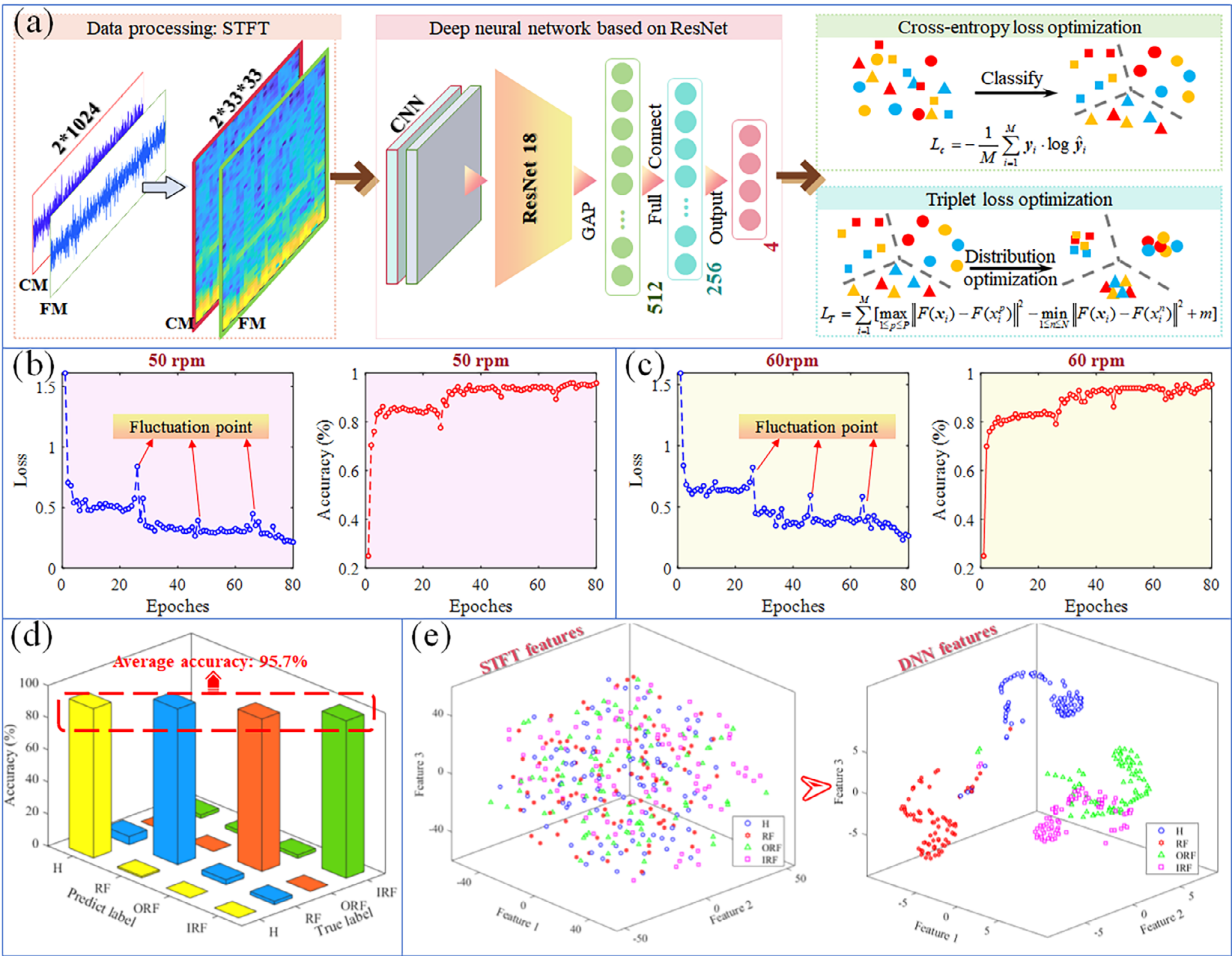
Wind turbine bearing fault diagnosis based on SST‐DTRB: a) Proposed DL‐based model, b) changes in loss and accuracy with training epochs at speeds of 50 and c) 60 rpm, d) confusion matrix, e) dimensionality reduction visualization of STFT and DNN features.

To achieve intelligent bearing fault diagnosis, this section introduces deep neural networks (DNNs) for constructing an intelligent diagnostic model. Pre‐fabricated fault experiments were conducted for the aforementioned fault types (roller, outer race, and inner race), simultaneously collecting triboelectric self‐sensing electrical signals from the CM and FM ends, with a sampling frequency of 3125 Hz and a sampling time of 100 s. Considering wind turbine speed variations, data were collected at two rotational speeds of 50 and 60 rpm, each for 100 s. As shown in **Figure** **5**a, the time‐domain voltage signals were converted into time‐frequency spectrograms using STFT. The spectrograms from the CM and FM ends were concatenated to represent their spatial relationships, forming a 3D spatial time‐frequency spectrum. Subsequently, a DNN was employed to identify the bearing faults using voltage signals at different rotational speeds. Compared to the widely used convolutional neural networks (CNNs), residual networks (ResNets) introduce residual connections that allow information to propagate directly between layers, providing more powerful feature extraction capabilities and improved convergence to optimal solutions.^[^ ^30^, ^31^, ^32^, ^33^ ^]^ Therefore, an 18‐layer residual network (ResNet18) was used as the feature extraction module.

A fully connected neural network (FCNN) was added as a classifier to output the diagnostic results. The FCNN consisted of three layers with 512, 256, and 5 neurons, respectively. The signal processing was performed using MATLAB R2016b and Python 3.9 software. During the training phase, 70% of the samples from each rotational speed and fault state were randomly selected as the training set, and the remaining 30% served as the test set to evaluate the diagnostic performance. The numbers of samples in the training and test sets for each rotational speed were 780 and 334, respectively, with each sample having a length of 1024 and a sampling frequency of 3125 Hz. The model was trained on a Windows 10 operating system using an Intel i5‐14600KF CPU and a GeForce GTX 4060Ti GPU. During model training, cross‐entropy loss was employed as the classification loss of the model, optimizing the model by minimizing the difference between the predicted results and actual labels. The cross‐entropy loss *L_c_* can be expressed as

(4) $$L_{c}=-\frac{1}{M}\sum_{i=1}^{M}y_{i}\cdot \log{\hat{y}}_{i}$$

where *M* is the batch size of the training data, **y** *_i_* is the actual label vector of the *i*‐th sample, and ${\hat{y}}_{i}$ is the output probability vector of the classifier after softmax activation.

However, cross‐entropy loss focuses solely on the classification performance of the model on the training set and cannot optimize the feature distribution, making the model more susceptible to local optima and poor robustness. Therefore, triplet loss was introduced to optimize feature distribution and guide model training, enabling the model to learn more discriminative and generalizable feature representations during the training process, thus improving diagnostic accuracy.^[^ ^34^, ^35^, ^36^ ^]^ The triplet loss *L_T_* can be expressed as

(5) $$L_{T}=\sum_{i=1}^{M}\left[\max_{1\leq p\leq P}{\left\|F\left(x_{i}\right)-F\left(x_{i}^{p}\right)\right\|}^{2}-\min_{1\leq n\leq N}{\left\|F\left(x_{i}\right)-F\left(x_{i}^{n}\right)\right\|}^{2}+m\right]$$

where *F*(∙) is the feature extraction module combined by ResNet18, **x** *_i_* represents the *i*‐th training sample, $x_{i}^{p}$ is a positive sample that has the same label with **x** *_i_*, *P* is the number of positive samples, $x_{i}^{n}$ is a positive sample which has the different label with **x** *_i_*, *N* is the number of negative samples, and *m* means the minimum distance boundary. According to parameter comparison analysis, *m* is set to 1.

Then, model training can be achieved by constructing a combined loss *L_tra_* based on the above cross entropy loss and triplet loss. *L_tra_* can be expressed as

(6) $$L_{tra}=L_{c}+\eta \cdot L_{T}$$

where *η* is the weight coefficient, determining the proportion of the triplet loss.

As shown in Figure 5b,c, the model achieved effective convergence at rotational speeds of 50 and 60 rpm. The fluctuation points in the loss allowed the model to escape the local optima during convergence and converge further. After 80 epochs of model training, the model gradually stabilized and the loss was reduced to its minimum value. As shown in Figure 5d, the confusion matrix demonstrates an average accuracy of 95. 6%, proving that the trained model effectively identified various faults in wind turbine DTRBs. Furthermore, as illustrated in Figure 5e, the t‐distributed stochastic neighbor embedding (t‐SNE) feature dimensionality reduction method confirms that the employed ResNet18 feature extraction module effectively transformed the original multi‐dimensional time‐frequency features into highly distinguishable features.^[^ ^37^, ^38^, ^39^ ^]^ In the 3D feature space, samples from various categories exhibited good clustering characteristics. The diagnosis model, trained with data at different rotational speeds, possesses good adaptability to various operating conditions. In practical applications, representative operating conditions and fault types in wind power should be fully considered before model training to ensure the model's generalization ability and robustness. Meanwhile, monitoring based on indicators such as skidding ratio can effectively prevent missed detection of new faults. In summary, this section has presented the implementation of a high‐precision fault diagnosis based on SST‐DTRB using a DNN. The model can adapt to the variable speed conditions of wind turbines, providing a solution for the intelligent monitoring and fault diagnosis of wind power equipment.

## 5 Conclusion

This study has presented the development of an in situ self‐powered sensing SST‐DTRB that integrates a TENG with a DTRB. The proposed SST‐DTRB design utilizes the axial clearance between the bearing rows to install a symmetrical single‐electrode TENG. The experimental results demonstrated the high sensitivity, stability, reliability, and robustness of the monitoring solution in material selection and design clearance, which is capable of long‐term operation without an external power source. The output of the SST‐DTRB can be enhanced by increasing the rotational speed, reducing the design clearance, and using PTFE as a dielectric ring material. Notably, triboelectric signals contain rich characteristic components, proving effective for detecting rotor conditions. The durability of the SST‐DTRB was validated through a 24‐h continuous operation test, confirming its reliability for long‐term applications. The effectiveness and self‐sensing capability of the SST‐DTRB at variable speeds were verified using a wind turbine test bench with a consistent drivetrain structure. By converting multi‐channel outputs into multi‐dimensional time‐frequency spectrograms and implementing fault diagnosis based on the DL‐based model, an accuracy of up to 95.6% was achieved under multiple operating conditions. The t‐SNE visualization confirmed good clustering characteristics among various fault categories. The SST‐DTRB provides real‐time self‐monitoring and fault diagnosis capabilities for wind turbines, offering new insights for the development of intelligent monitoring systems for renewable energy equipment. Future research will focus on integrating SST‐DTRB with other energy harvesting technologies and extending its application to high‐speed scenarios.

## 6 Experimental Section

### Fabrication of SST‐DTRB

The SST‐DTRB structure primarily consisted of inner and outer races, rollers, a cage, a PTFE dielectric layer, electrode plates, and locating pins. This design innovatively combined a TENG with a traditional DTRB structure, achieving integrated, self‐powered, self‐sensing, and fault diagnosis functionality for the DTRB. Notably, by leveraging the unique separable symmetrical structure of the tapered rollers of the DTRB, electrode plates were installed on the axially symmetrical surface of the outer race and secured using locating pins. Two dielectric rings were adhered to the end faces of the cage, rotated with it, and periodically contacted the electrode plates to generate TENG electrical signals. Specifically, the SST‐DTRB adopted a single‐electrode mode TENG. Compared with the traditional free‐mode TENG, the single‐electrode mode TENG halved the number of connections, thereby reducing the installation and maintenance complexity.

### Establishment of SST‐DTRB Test System

The bearing used in the experiment was a DTRB from Harbin Bearing Manufacturing Co., Ltd., which is a typical representative double‐row tapered roller bearing made of high‐quality bearing steel. It could simultaneously bear radial and axial loads, making it suitable for applications such as wind turbine main shafts that experience combined loads and heavy‐duty environments. A Kesley 6514 electrometer was used to measure the open‐circuit voltages and short‐circuit currents. The output voltage readings were acquired using an NI 9222 analog‐to‐digital converter, with a four‐channel voltage measurement module and a voltage range of ±10 V.

### Signal Processing and Model Training

Triboelectric self‐sensing signals from the CM and FM ends were collected simultaneously at a sampling frequency of 3125 Hz. Considering wind turbine speed variations, data were collected at two rotational speeds, 50 and 60 rpm, each for 100 s. The signal processing was performed using MATLAB R2016b and Python 3.9 software. During the training phase, 70% of the samples from each rotational speed and fault state were randomly selected as the training set, and the remaining 30% served as the test set to evaluate the diagnostic performance. The numbers of samples in the training and test sets for each rotational speed were 780 and 334, respectively, with each sample having a length of 1024 and a sampling frequency of 3125 Hz. The model was trained on a Windows 10 operating system using an Intel i5‐14600KF CPU and GeForce GTX 4060Ti GPU. To optimize the model and prevent overfitting, a K‐fold cross‐validation strategy (K = 5) was implemented for hyperparameter selection, dividing the dataset into 5 parts and training the model on different combinations to ensure robust performance across varied data distributions. Additionally, Dropout layers with a rate of 0.3 were inserted after each fully connected layer in the classifier, randomly deactivating neurons during training to prevent overfitting and enhance generalization capability. During training, the batch size was 64, the learning rate was 0.001, and the Adam optimizer was used.

## Conflict of Interest

The authors declare no conflict of interest.

## Data Availability

The data that support the findings of this study are available from the corresponding author upon reasonable request.
